# A Computer-Aided Diagnosis System for Dynamic Contrast-Enhanced MR Images Based on Level Set Segmentation and ReliefF Feature Selection

**DOI:** 10.1155/2015/450531

**Published:** 2015-01-06

**Authors:** Zhiyong Pang, Dongmei Zhu, Dihu Chen, Li Li, Yuanzhi Shao

**Affiliations:** ^1^School of Physics and Engineering, Sun Yat-sen University, Guangzhou 510275, China; ^2^Imaging Diagnosis and Interventional Center, Cancer Center, The Sun Yat-sen University, Guangzhou 510060, China

## Abstract

This study established a fully automated computer-aided diagnosis (CAD) system for the classification of malignant and benign masses via breast magnetic resonance imaging (BMRI). A breast segmentation method consisting of a preprocessing step to identify the air-breast interfacing boundary and curve fitting for chest wall line (CWL) segmentation was included in the proposed CAD system. The Chan-Vese (CV) model level set (LS) segmentation method was adopted to segment breast mass and demonstrated sufficiently good segmentation performance. The support vector machine (SVM) classifier with ReliefF feature selection was used to merge the extracted morphological and texture features into a classification score. The accuracy, sensitivity, and specificity measurements for the leave-half-case-out resampling method were 92.3%, 98.2%, and 76.2%, respectively. For the leave-one-case-out resampling method, the measurements were 90.0%, 98.7%, and 73.8%, respectively.

## 1. Introduction

Because early detection of breast cancer offers the best chance for a cure, regular screening has been identified as a key to improving breast cancer survival rates. Breast cancer is commonly based on X-ray mammography and ultrasound, which have a low sensitivity and are not effective in dense breast tissue. Dynamic contrast-enhanced (DCE) magnetic resonance imaging (MRI) has been shown to be the most sensitive screening methodology for the detection of invasive breast cancer and can detect breast cancer missed by mammography [1, 2]. Computer image analysis provides various techniques for analyzing medical images. Computerized methods have recently shown great potential for providing radiologists with a second opinion about the visual diagnosis of the malignancy of mammographic masses.

However, compared with mammography, relatively fewer computer-aided diagnosis (CAD) systems have been developed specifically for breast MRIs (BMRIs). Most CAD systems require radiologists to manually (or semiautomated) segment tumors from the imaging data [3–6]. As a result, there is an urgent need to develop a fully automated CAD system that allows radiologists to diagnose the data more efficiently. Three key components of such an automated CAD system commonly include an appropriate segmentation algorithm, an appropriate feature extraction algorithm, and an appropriate classification algorithm responsible for the differential diagnosis of malignant and benign masses.

DCE-MRI automated segmentation should include breast and mass segmentation. However, few studies have focused on breast segmentation. Several mass segmentation methods such as the threshold, region growing, clustering, and 3D level set (LS) methods have been proposed. Shi et al. [6] used the fuzzy *c*-mean (FCM) clustering algorithm followed by a 3D LS method to refine segmentation. Region growing methods [7] gather pixels or subregions from larger regions using predetermined similarity criteria, which suffer from sensitivity to the selection of initial seed points. Among the clustering techniques, the FCM [8] has received much attention, but it exhibits a low performance [9] owing to its oversensitivity to noise. Liney et al. [10] presented a user-interaction-threshold method to extract the region of interest (ROI), a method that requires manual intervention. A novel two-step approach that incorporates FCM clustering and a gradient vector flow (GVF) snake algorithm for mass contour segmentation in BMRIs was also designed and obtained encouraging results [3].

Features can be extracted from original ROIs, segmented masses, and patient information. Most CAD systems in the literature have explored morphological and texture features based on segmented masses. As adjunct diagnostic criteria, the morphological features of BMRIs have proven useful in improving specificity without significantly decreasing sensitivity [3, 11–13]. Texture analysis (i.e., homogeneity and regularity with diagnosis potential in MRIs) is significantly associated with breast tumor subtype and neoadjuvant therapy response and has been used extensively to quantify MRI characteristics [3, 14–16]. A preliminary study [3] proved the potential discriminatory power of the image features estimated from both morphological and texture features.

Feature selection, which refers to the choosing of a subset of attributes from a set of original attributes, is an important issue in building classification systems. However, few studies have investigated the feature selection performance of both morphological and texture features in discriminating pathologically verified breast masses.

Classifier selection is a crucial step for computerized classification of malignant and benign breast masses. Classical breast CAD classification algorithms include the support vector machine (SVM) [17], naive Bayes (NB) classifier technique [18], *k*-nearest neighbors (KNN) [18], logistic regression (LR) model [19], and linear discriminant method (LDA) [20]. SVMs have been shown to outperform many alternative pattern-recognition techniques for breast cancer detection from MRI [5, 21, 22]. The preliminary study [3] systematically investigated diagnostic performance by combining the merits of both morphological and texture features using the Fisher stepwise discriminant analysis model.

In this study, a fully automated DCR-MRI CAD system is developed, in which a fully automated breast segmentation algorithm based on curve fitting is proposed. The Chan-Vese (CV) model and LS method are used to evolve the segmentation of breast masses. Both the morphological and texture features of a BMRI mass are calculated based on the proposed computerized segmentation contour and radiologists' delineation, respectively. To remove redundancy and increase the diagnostic capabilities of the features, a ReliefF algorithm [23], which is one of the most successful feature selection algorithms, is adopted to select and optimize the features. SVMs have been used to diagnose breast cancer and achieved the highest classification accuracy among the available artificial intelligent methods. This study focuses on evaluating the SVM as a potential classifier in combination with ReliefF feature selection to classify benign and malignant masses. The computational results of both the segmentation and characterization of breast masses are compared via manual delineation and the pathological results given by experienced radiologists.

## 2. Materials and Methods

**Figure 1:**
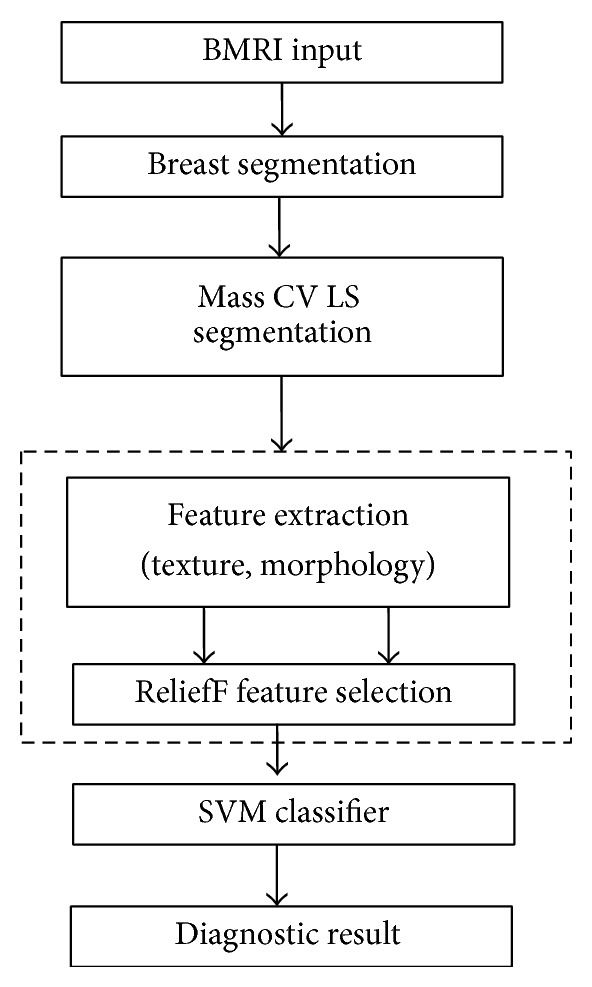
Flowchart of computerized mass segmentation and characterization in a BMRI.

Figure 1 shows a flowchart of the main steps of the proposed DCE-MRI CAD system. All five steps were fully automated. The first step was to segment the breast as an organ from other parts in the BMRI via preprocessing to identify the air-breast interfacing boundary and curve fitting for the chest wall line (CWL) segmentation. Second, the segmented breast was processed further by applying the LS model to obtain the final mass segmentation. Morphological and texture features were extracted from the LS segmentation in the computerized characterization section. A ReliefF algorithm has been successfully used in many large subset feature selection tasks, and here it was guided to estimate the weight of the morphological and texture feature. It was fundamentally important to select the relevant and necessary features in the preprocessing step. Finally, a SVM classifier was used to evaluate the ability of the mass descriptors to discriminate the different ROIs to determine whether they represented malignant or benign masses.

### 2.1. Breast Mass Database

The dataset consisted of 120 female patients (42 benign and 78 malignant) who had been examined with a final histopathology confirmation (age range = 29–66 years, mean age = 47.5 years) from Sun Yat-sen University Cancer Center (Guangzhou, China). Patients with suspicious breast masses were recruited with written informed consent. The Ethics Committee of Sun Yat-sen University Cancer Center approved the study. Patients were scanned in the prone position using a 1.5 T superconductive magnetic system (GE, Signa, HDx) with a breast-specific 4-channel phased-array surface coil. The patients were injected with a contrast medium using a hand venipuncture technique and then scanned in the prone position with the bilateral breast naturally hanging into the two holes of the coil and their feet placed into the machine. The patients had not received treatment before nuclear magnetic detection. In this study, only mass-like masses that showed strong contrast enhancements were selected. The database of the images for each case included one sagittal postcontrast image slice that showed an obvious contrast enhancement and demonstrated the maximum dimension of a mass. All of the images in this dataset were 512 × 512 pixels in size and had an 8-bit gray-level resolution [3].

### 2.2. Breast Segmentation

**Figure 2:**
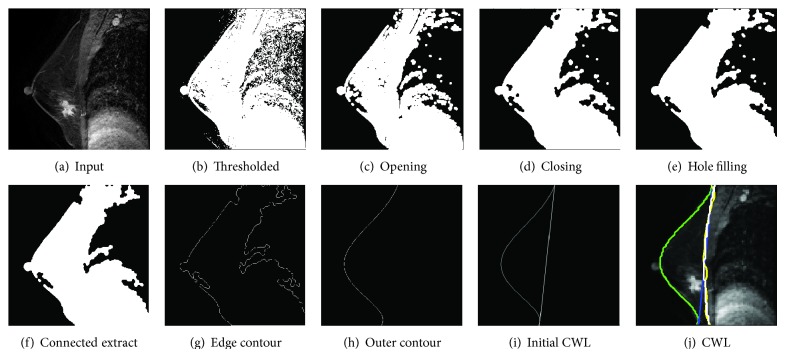
Processing steps for breast segmentation.

Breast segmentation is performed to separate the breast as an organ from the chest wall, a critical and challenging first step in automated BMRI analysis. For this task, the segmentation algorithm requires the identification of both the air-breast interface and the CWL. Although breast segmentation in BMRIs is an important topic for cancer treatment and diagnosis, it is mostly performed using a manual or semiautomated delineation method [3, 24, 25]. Few automated methods have reportedly been used in an MRI CAD system [26–28]. These methods require a large number of training samples or complex calculation. The segmentation method used in this study consisted of a preprocessing step to identify both the air-breast interfacing boundary and curve fitting for the CWL segmentation. The air-breast interface is initially identified given a BMRI scan sagittal postcontrast image. The air-breast boundary is relatively easy to identify in a BMRI due to the highly intense contrast of the boundary. Preprocessing techniques including thresholding (image binarization), image morphological opening, morphological closing, hole filling, connected components extraction, and edge contour extraction were applied sequentially to each 2D slice (Figure 2). Once the air-breast interface was identified, there were three main steps to refining the breast segmentation: (1) using the curve fitting method to fine fit the outline of the breast; (2) drawing a straight line according to the outer contour line of the two vertices as the initial CWL; and (3) adopting the GVF snake iteration approach and curve fitting iteration to increase the accuracy of the CWL edge extraction.  Figure 2(h) shows the result of the outer contour.  Figure 2(i) shows the result of using the two vertices to draw a straight line as the initial CWL. The straight line based on the GVF snake iterative algorithm is shown in yellow in Figure 2(j). The green line represents the fitting of the breast contour line, with the polynomial fitting method used to obtain the CWL.

### 2.3. Mass Segmentation

Because breast masses on DCE-MRI scans may be more pronounced at the periphery than the internal region of the mass, the object segmented by the FCM clustering algorithm may contain holes. The preliminary study [3] implemented a novel two-step approach that incorporated FCM clustering and a GVF snake algorithm for mass contour segmentation on a BMRI. Although the snake model allows for fast evaluation, it makes handling topological changes difficult. The main drawbacks of the snake algorithm are its sensitivity to initial conditions and the difficulties associated with topological transformations. Moreover, the snake segmentation method of a contour is too smooth and has a disadvantageous influence on the subsequent characterization and differentiation of benign and malignant breast masses. In this study, an LS-based method was adopted to produce a refined ROI. An FCM-based method was not used to produce an initial segmentation of the ROI. FCM initial segmentation has been proven not to improve performance. A current implementation of the GVF snake was compared with a previous implementation. The LS segmentation method used is detailed as follows.

The LS method is a deformable model that can capture object's shape or surface by numerically solving a well-designed partial differential equation (PDE). The LS method has increasingly been applied to image segmentation in the past decade, as it allows for cusps, corners, and automatic topological changes such as object splitting and merging. It has several advantages over its predecessor, the explicit active contour model. The curve is represented implicitly via a Lipschitz function *ϕ* in level *C* = {(*x*, *y*)∣*ϕ*(*x*, *y*) = 0}, and the evolution of the curve is given by the zero-level curve at time *t* of the function *ϕ*(*x*, *y*, *t*). The isocontour *ϕ*(*x*, *y*) = 0, which encloses a region *Ω*, has specifically been referred to as the zero LS in the literature. Evolving the curve *C* in a normal direction at a speed *F* amounts to solving the differential equation. Consider

(1) $$\begin{array}{c} \frac{\partial \phi }{\partial t}=\left|\nabla \phi \right|F,\;\;\phi \left(x,y,0\right)=\phi_{0}\left(x,y\right). \end{array}$$

In the LS formulation of the model, *C* ⊂ *Ω* is represented by the zero LS of a Lipschitz function *ϕ* : *R* → *Ω*, such that

(2) C=∂ω=x,y∈Ω:ϕx,y=0,insideC=ω=x,y∈Ω:ϕx,y>0,outsideC=Ω∖ϖ=x,y∈Ω:ϕx,y<0.

Because the classical LS models rely on the edge function (depending on the image gradient) to stop the curve evolution, they can detect only objects with edges defined by gradients. In practice, the discrete gradients are bounded. The stopping function is never zero on the edges, and the curve may pass through the boundary, especially for the models used in [29].

Chan and Vese [30] proposed a new model for active contours to detect objects in a given image based on curve evolution techniques, the Mumford-Shah functional for segmentation, and LSs. The Chan-Vese model can detect objects whose boundaries are not necessarily defined by gradients.

Define the evolving curve *C* in *Ω* as the boundary of an open subset *ω* of *Ω* (i.e., *ω* ⊂ *Ω* and *C* = ∂*ω*). In what follows, inside (*C*) denotes the region *ω* and outside (*C*) denotes the region *Ω*∖*ϖ*.

The Mumford-Shah functional for segmentation is

(3) FMSu,C=μ LengthC+λ∫Ωu0x,y−ux,y2dx dy +∫Ω∖C∇ux,y2dx dy,

where *u*_0_ is a given image and *μ* and *λ* are positive parameters. The solution image *μ* obtained by minimizing this functional is formed by smooth regions denoted by *R*_*i*_ and sharp boundaries denoted by *C*.

The energy functional of the Chan-Vese model is defined by

(4) Fc1,c2,C=μ·LengthC+v·AreainsideC +λ1∫insideCμ0x,y−c12dx dy +λ2∫outsideCμ0x,y−c22dx dy,

where *μ* > 0, *v* > 0, *λ*_1_ > 0, *λ*_2_ > 0 are fixed parameters.

The Heaviside function *H* and one-dimensional Dirac measure *δ*_0_ are, respectively, defined as

(5) $$\begin{array}{c} H\left(Z\right)=\left\{\begin{array}{cc} 1, & z\geq 0,\\ 0, & z<0, \end{array}\right.\;\;\delta_{0}\left(z\right)=\frac{d}{dz}H\left(Z\right). \end{array}$$

For the LS formulation of the variational active contour model, the Chan-Vese model replaces the unknown variable *C* with the unknown variable *ϕ*. The energy *F*(*c*_1_, *c*_2_, *ϕ*) can then be written as

(6) Fc1,c2,ϕ  =μ·∫Ωδϕx,y∇ϕx,ydx dy   +v·∫ΩHϕx,ydx dy   +λ1∫Ωμ0x,y−c12Hϕx,ydx dy   +λ2∫Ωμ0x,y−c221−Hϕx,ydx dy,

(7) $$\begin{array}{c} H_{2,\varepsilon }\left(z\right)=\frac{1}{2}\left(1+\frac{2}{\pi }\arctan\left(\frac{z}{\varepsilon }\right)\right). \end{array}$$

The Dirac delta function *δ*, which is the derivative of the Heaviside function *H*, is accordingly replaced by the derivative of *H*_*ε*_, which is calculated as

(8) $$\begin{array}{c} \delta_{2,\varepsilon }\left(z\right)=H_{2,\varepsilon }^{\prime }\left(z\right)=\frac{1}{\pi }\frac{\varepsilon }{\varepsilon^{2}+z^{2}}. \end{array}$$

Denote by *F*_*ε*_ the associated regularized functional, defined as

(9) Fεc1,c2,ϕ  =μ·∫Ωδεϕx,y∇ϕx,ydx dy   +v·∫ΩHεϕx,ydx dy   +λ1∫Ωμ0x,y−c12Hεϕx,ydx dy   +λ2∫Ωμ0x,y−c221−Hεϕx,ydx dy.

Keeping *c*_1_ and *c*_2_ fixed and minimized in terms of *F*_*ε*_, the associated Euler-Lagrange equation can be deduced for *ϕ*. Consider

(10) ∂ϕ∂t=δεϕμdiv⁡∇ϕ∇ϕ−v−λ1μ0−c12hhhihhhh+λ2μ0−c22∇ϕ∇ϕ,ϕ0,x,y=ϕ0x,y.

Formulations (7), (8), (9), and (10) are numerical approximations of the CV LS model.

**Figure 3:**
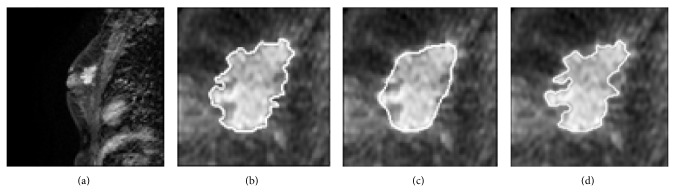
An example of DCE-MRI mass segmentation: (a) original image; (b) initial segmentation result on using the FCM-based method; (c) deformation of GVF snake using FCM-based contour for initialization; (d) LS segmentation result.

The mass extracted by FCM-based segmentation, FCM-GVF, and LS was compared with the reference standard, that is, the radiologist's manual segmentation.  Figure 3(a) shows an example ROI that contains a mass proven to be a malignant tumor via biopsy. The mass edge is blurred and partially overlapped by other soft tissues, so the traditional segmentation methods are prone to segmentation leak.  Figure 3(b) shows the boundary resulting from the FCM clustering and morphological opening. Although the FCM segmentation covers most of the mass edges visually, it is still slightly undersegmented on the lower right corner of the mass. The boundary was then refined by the GVF snake segmentation as shown in Figure 3(c) and by the LS segmentation as shown in Figure 3(d), respectively. In both figures, although the FCM-GVF segmentation covers most of the mass edges visually, it is still slightly too smooth. The LS segmentation covers most of the mass edges visually, allowing for cusps, corners, and automatic topological changes.

### 2.4. Feature Extraction and Selection

#### 2.4.1. Morphological and Texture Features

The morphological and texture features are the most commonly used features of a breast cancer CAD system. The CAD system in this study directly obtained the two features and required no other software. Texture is an intrinsic characteristic of an object and is important for medical image analysis [31]. Researchers have proposed various textural algorithms such as fractal-based description, texture spectrum, and the Markov random field model [32–34]. The gray-level cooccurrence matrix (GLCM) texture method has been investigated heavily since its introduction by Haralick et al. in 1973 and has demonstrated considerable promise in MRI texture analysis. Important texture information exists in the tissue surrounding a mass margin. In this study, 13 textural measures were calculated for the nearest pixels (distance: 1 pixel) in 4 limited directions: 0°, 45°, 90°, and 135°, respectively. Thirteen features including the angular second moment, contrast, correlation, inverse difference moment, sum average, sum variance, sum entropy, entropy, difference average, difference variance, difference entropy, information measure of correlation 1, and information measure of correlation 2 were calculated from the GLCM. Owing to the isotropic texture of the images investigated, the features evaluated in the current study were the averages over the four directions. These texture features contained some important information about homogeneity, contrast, and other organized image structures.

In addition to the texture feature, eight morphological features were selected and calculated to describe the morphological properties as defined in the breast imaging reporting and data system lexicon. These features included compactness, spiculation, extent, elongation, solidity, circularity, entropy of radial length distribution, and eccentricity. A detailed description of these features can be found in the preliminary study [3].

#### 2.4.2. Feature Selection

Feature selection [17, 35–39] is used to identify and remove as much irrelevant and redundant information as possible. It can improve the accuracy of the resulting model and decrease the calculation time of the induction algorithm. In this study, texture and morphological feature subset selection was used to find the set of features that best distinguished malignant from benign masses.

Feature subset selection research has traditionally looked at relevant features. Feature selection algorithms usually fall into two categories [35]: the filter and wrapper methods. Although the wrapper has the advantage of better performance, its usage in the biomedical arena is limited due to its high computational cost [35]. A filter algorithm was used in this study to alleviate this problem. Relief [39] is a well-known filter algorithm that estimates the quality of attributes according to how well their values distinguish between close instances. However, Relief is ineffective at removing redundant features, as two predictive but highly correlated features are probably both highly weighted. ReliefF [23] extends relief, enabling the method to work with noisy and incomplete datasets and to deal with multiclass problems. ReliefF is a simple yet efficient procedure used to estimate the quality of attributes in problems with strong dependencies between attributes. In practice, ReliefF is usually applied independently of the chosen predictor in data preprocessing as a feature subset selection method. The key idea of ReliefF is to estimate the quality of attributes according to how well their values distinguish between close instances. Given a randomly selected instance *R*_*i*_ from class *L*, ReliefF searches for *k* of its nearest neighbors from the same class, known as nearest hits *H*, and also *k* of its nearest neighbors from each of the different classes, known as nearest misses *M*. It updates the quality estimation *W*(*F*) for all attributes *F* depending on their values for *R*_*i*_, hits *H*_*j*_, and misses *M*_*j*_(*C*). The updated average of the contribution of all of the hits and misses can be calculated via the following equation:

(11) WF =WF−∑j = 1kdiffF,Ri,Hjm·k  +∑C≠classP∑j=1k2PC1−PclassRihhhhhhhhhhhh×∑j = 1kdiff(F,Ri,MjC)×m·k−1,

where Function diff(*F*; *I*1; *I*2) calculates the difference between the attribute *F* values for two instances *I*1 and *I*2. The contribution for each class of misses is weighted with the prior probability of that class *P* (*C*) (estimated from the training set). 1 − *P* (class(*R*_*i*_)) represents the sum of the probabilities for the miss classes.

**Figure 4:**
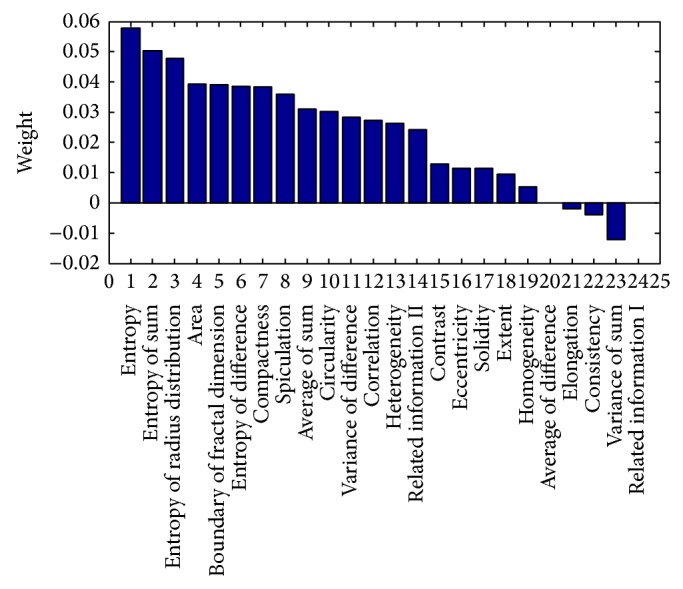
Weights calculated by ReliefF for morphological and texture features.

In this study, ReliefF was applied to find a candidate feature subset from the available morphological and texture features. The parameters of the weight distribution histogram were obtained as shown in Figure 4. The weight coefficient range was [−1, 1], with values closer to 1 indicating a stronger classification ability. The model with the features selected from the ReliefF feature selection methods was tested on the SVM classifier. Eight higher weight features were used to classify benign and malignant breast masses according to the weight distribution of the characteristic parameters.

### 2.5. Classification

Once the features were extracted and selected from the segmented masses, the data with *n* selected features could be fed into an appropriate classification model. The literature has discussed many different approaches to diagnosing breast cancer, such as SVM, LDA, NB, KNN, and ANN. SVMs have been used to diagnose breast cancer and achieved the highest classification accuracy among the available artificial intelligent methods. Therefore, in this study, an SVM classifier was used to evaluate the diagnostic performance of carefully selected variables. The Fisher classifier was used for comparison, as the preliminary study [3] found that it was more generalizable for unknown cases than other more complex classifiers given a limited training sample size.

The SVM was developed by Vapnik [40] based on Vapnik-Chervonenkis (VC) theory and the structural risk minimization (SRM) principle and has been used for many machine learning tasks such as pattern recognition, object classification, and regression analysis. It seeks a tradeoff between minimizing the training set error and maximizing the margin to achieve a high level of generalization and remain resistant to overfitting. In addition, SVMs have a strict theory and mathematical foundation that presents no local optimization or dimensional problems. Chang and Lin [41] developed LIBSVM, which was implemented for the purposes of this study. There were two steps involved in the LIBSVM implementation: (1) the dataset was trained to obtain a model and (2) the model was used to predict the information for the testing dataset. The final output was the classification accuracy for breast cancer prognosis, which classified the patients as having a malignant or benign diagnosis with the optimum feature of the subset.

## 3. Results and Discussion

### 3.1. Segmentation Performance

Automated breast mass segmentation is an important step for the CAD system. The accurate delineation of masses in a BMRI is crucial for diagnosis and the associated image-guided biopsy.  Table 1 summarizes the mean values and standard deviations of the areas from the mass contours, which were segmented by the FCM-based method, FCM-GVF method, LS method, and radiologists' manual delineation, respectively. The differences between the computerized method and radiologists' manual delineation were analyzed using Pearson's correlation coefficient (Pearson's *r*) and paired Student's *t*-test (Table 1). According to the original hypothesis, there is no significant difference between the two groups of mass areas segmented by different methods. Pearson's correlation coefficient was used to measure the correlation between the computer segmentation and reference standard. The paired Student's *t*-test was used to evaluate the significance of the differences between the segmentation.

Pearson's *r* between the mass areas segmented by the FCM-based method and the radiologists' manual delineation was 0.9807, and the paired *t*-test between the areas extracted by the two methods achieved a *P* value of 0.7173. This indicates that the areas worked out by the two methods were highly correlated without a significant difference in averages. After the *r* and *P* values were refined using the GVF and LS methods, they increased and continued to show a high correlation between the areas without a significant difference in average (*P* > 0.05). This indicates that the three computerized methods could help radiologists achieve accurate delineation. The LS method showed the best performance among the three methods.

**Figure 5:**
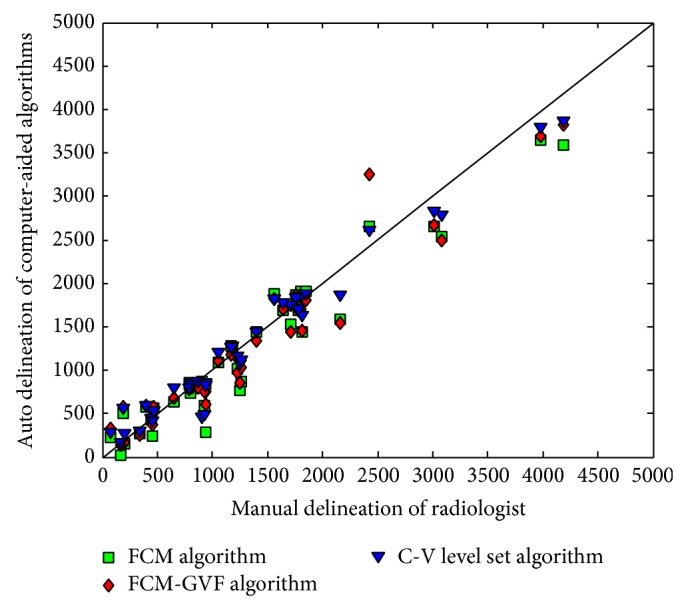
Scatter plot of the mass areas segmented by computerized and radiologists' manual delineation. The diagonal line represents the most perfect segmentation performance. The squares represent areas segmented by the FCM-based initial method. The diamonds represent areas extracted using the FCM-GVF method. The triangles represent areas extracted using the LS method.

Figure 5 shows the log-log scatter plot of the areas measured using the computerized method versus radiologists' manual segmentation. The mass area is calculated by the number of pixels in the mass region. The log-log scatter plot was drawn because the mass area had a wide range. Judging by the distribution of the data points in Figure 5, the computerized methods somewhat underestimated the mass area compared with the radiologists' reference area, as most of the data points are distributed below the reference diagonal line. The FCM-GVF method had a smaller underestimation than the FCM-based method. One drawback of the FCM-based method is that it depends simply on intensity information and does not include the spatial relationships of pixels. For a more complicated mass enhancement, it is difficult for the FCM-based method to locate the contour that approaches near to the realistic mass contour. The FCM-GVF method improves the initial segmentation when deforming to a balance of internal and external forces. However, one drawback of the FCM-GVF method is that it depends on image edge information. The CV LS method can detect objects whose boundaries are not necessarily defined by gradients. In this study, it showed a densely distributed scatter along the diagonal. As such, its segmentation results (i.e., better segmentation) approximated those of the radiologists' hand-painted results.

**Figure 6:**
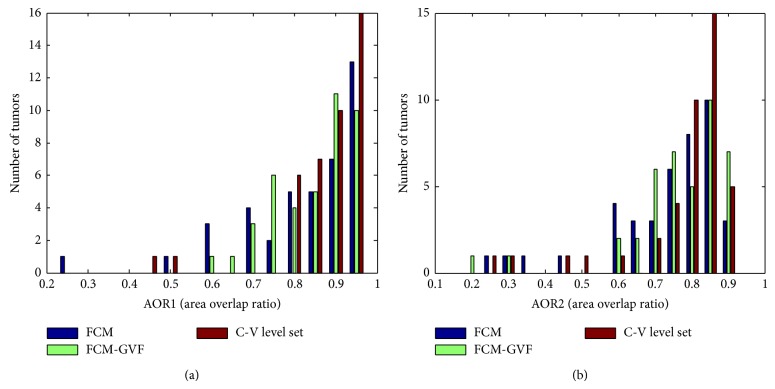
Histograms of the overlap measures for the computerized methods: (a) AOR1; (b) AOR2. The closer the AOR value is to one, the better the segmentation performed. The LS method exhibited the best performance of the three methods.

Figure 6 exhibits the histograms of the overlap measures for the FCM-based, FCM-GVF, and LS methods. All of the masses segmented using the three methods have values of AOR1 and AOR2, with the most concentrated distribution over 0.6. Figure 6 also shows that the bars of the LS method are denser from 0.7 to 1. These results indicate that the LS automated segmentation method performed better for the masses.

### 3.2. Feature Select and Classification Performance

The preliminary study [3] proved that morphological and texture features can be used to classify breast masses and that the features of the computerized segmentation method can provide a more efficient and objective diagnostic performance when discriminating between benign and malignant masses. This study sought to verify whether the features of LS computerized segmentation and the ReliefF feature selection method can work together to improve diagnostic performance when discriminating between benign and malignant masses. Two classification methods including the Fisher and SVM methods were experimented with and their results were subsequently compared. The Fisher classifier was chosen because the preliminary study [3] found it to be more generalizable to unknown cases than other more complex classifiers when the training sample size is limited. SVMs have been used to diagnose breast cancer and have achieved the highest classification accuracy among the available artificial intelligent methods according to the literature. In the current study, a two-loop leave-one-case-out resampling procedure was designed to train the Fisher and SVM classifiers and test performance using *N* available cases, where *N* = 120.

To verify the classification accuracy of the newly developed CAD system, a subset of eight features whose weights ranked at the top in the ReliefF algorithm was selected as an independent test set. In addition, as these eight features could form two hundred and fifty-five different cases, all of the cases were tested and the optimal classification result was chosen as the independent experimental result. The corresponding selected features are shown in Tables 2 and 3.

As shown in Tables 2 and 3, when all eight of the features, including entropy, entropy of sum, entropy of radius distribution, area, boundary of fractal dimension, entropy of difference, compactness, and speculation, were selected as a subset for the experiment, their accuracy, sensitivity, and specificity varied among the different segmentation and classification methods. The two tables make it clear that the combination of the CV LS segmentation method and SVM classifier achieved the best performance out of all of the methods. The leave-half-case-out test in Table 2 exhibits an accuracy level of 92.3%, a sensitivity level of 98.2%, and a specificity level of 76.2%. The leave-one-case-out test in Table 3 exhibits an accuracy level of 90.0%, a sensitivity level of 98.7%, and a specificity level of 73.8%.

### 3.3. Discussion

The results of our experiment demonstrate that our new DCE-MRI CAD system using CV LS/ReliefF/SVM hybrid model exhibited the best diagnostic performance. Nowadays, our CAD system has shown that the eight morphological and texture features in breast MRI as adjunct diagnostic criteria can improve the specificity without significantly reducing the sensitivity. The most widespread CAD applications in the breast mostly will take into account dynamic features. For example, Baltzer et al. [42] have shown that fast visual assessment of dynamic data using CAD calculated parametric images is feasible without a decrease in diagnostic accuracy. They also proved that the combination of multiple dynamic and morphological MRI criteria seems to have the potential for a differential diagnosis of inflammatory breast carcinomas and acute mastitis [43]. Our future work will evaluate whether a combination with dynamic features evaluation could further improve our CAD system diagnostic accuracy.

## 4. Conclusion

This study developed a fully automated BMRI prognostic system that implemented breast segmentation, tumor segmentation, feature extraction, feature selection, and classification between benign and malign tumors. Compared with the FCM and GVF snake segmentation methods, the segmentation performance indicated that the CV LS computerized segmentation method is a more accurate method for automatically determining a suspicious mass region and can help radiologists in their detection and delineation of BMRIs. The ReliefF algorithm was useful in selecting an optimal subset of breast tumor features. The subset could be used to decrease feature dimensions and weight minimum distance classifiers. In terms of computerized characterization, the Fisher and SVM methods were used separately to select morphological and texture features and make classifications with the adoption of a leave-one-case-out cross-validation method and a leave-half-case-out validation method. In conclusion, the ReliefF/SVM/CV LS hybrid model exhibited the best performance (accuracy = 90.0%, sensitivity = 98.7%, and specificity = 73.8% for the leave-one-case-out validation; accuracy = 92.3%, sensitivity = 98.2%, and specificity = 76.2% for the leave-half-case-out validation). The new DCE-MRI CAD system may assist radiologists in delineating and characterizing BMRI masses, for example, by quantifying morphological and texture features and characterizing DCE-MRI masses as malignant or benign. It also has the potential to assist radiologists in decreasing the biopsy rate without increasing false negatives.

Additional tests and experiments must be conducted to further verify the results obtained in this study. Future work could increase the sample size of the dataset by providing more medical samples to reflect the real population. A much greater effort will be required to design effective computer-vision methods that can fully exploit the image information in DCE-MRIs to improve segmentation and feature selection.

## Figures and Tables

**Table 1:** Areas, statistical comparisons, and area overlap measures of computerized and radiologists' manual delineation.

Segmentation method	Area (mean ± SD pixels)	Pearson's correlation	*t*-test *P* value	AOR1 (mean ± SD)	AOR2 (mean ± SD)
FCM	1,439.5 ± 1,300.7	0.9807	0.7173	0.84 ± 0.14	0.75 ± 0.15
GVF-snake-FCM	1,474.7 ± 1,333.9	0.9828	0.8098	0.87 ± 0.09	0.78 ± 0.14
CV-level set	1,526.4 ± 1,334.8	0.9868	0.9449	0.89 ± 0.10	0.79 ± 0.14
Radiologists' manual	1,547.1 ± 1,380.5	—	—	—	—

**Table 2:** Classification results of the different segmentation methods (leave-half-case-out).

Segmentation method	Classification model	Accuracy (%)	Sensitivity (%)	Specificity (%)
FCM	Fisher	79.5	87.7	57.1
	SVM	74.4	82.5	52.4

FCM-GVF-snake	Fisher	80.8	86.0	66.7
	SVM	82.1	86.0	71.4

CV-level set	Fisher	91.0	96.5	76.2
	SVM	92.3	98.2	76.2

**Table 3:** Classification results of the different segmentation methods (leave-one-case-out).

Segmentation method	Classification model	Accuracy (%)	Sensitivity (%)	Specificity (%)
FCM	Fisher	83.3	91.0	69.0
	SVM	80.8	93.6	57.1

FCM-GVF-snake	Fisher	78.3	82.1	71.4
	SVM	82.5	93.6	61.9

CV-level set	Fisher	90.8	94.9	83.3
	SVM	90.0	98.7	73.8
